# *Nigella sativa* seeds mitigate the hepatic histo-architectural and ultrastructural changes induced by 4-nonylphenol in *Clarias gariepinus*

**DOI:** 10.1038/s41598-023-30929-w

**Published:** 2023-03-13

**Authors:** Mahmoud Abd-Elkareem, Alaa El-Din H. Sayed, Nasser S. Abou Khalil, Mohamed H. Kotob

**Affiliations:** 1 Cell and Tissues Department, Faculty of Veterinary Medicine, Assiut University, Assiut, 71526 Egypt; 2 Zoology Department, Faculty of Science, Assiut University, Assiut, 71516 Egypt; 3 Molecular Biology Researches & Studies Institute, Assiut University, Assiut, 71516 Egypt; 4 Department of Medical Physiology, Faculty of Medicine, Assiut University, Assiut, Egypt; 5 Department of Pathology and Clinical Pathology, Faculty of Veterinary Medicine, Assiut University, Assiut, Egypt

**Keywords:** Zoology, Biomarkers

## Abstract

Due to its prevalence in aquatic environments and potential cytotoxicity, 4-nonylphenol (4-NP) has garnered considerable attention. As a medicinal plant with numerous biological activities, *Nigella sativa* (black seed or black cumin) seed (NSS) is widely utilized throughout the world. Consequently, this study aimed to examine the potential protective effects of NSS against 4-NP-induced hepatotoxicity in African catfish (*Clarias gariepinus*). To achieve this objective, 18 fish (351 ± 3 g) were randomly divided into three equal groups for 21 days. The first group serves as a control which did not receive any treatment except the basal diet. The second and third groups were exposed to 4-NP at a dose of 0.1 mg L^−1^ of aquarium water and fed a basal diet only or supplemented with 2.5% NSS, respectively. The histological, histochemical, and ultrastructural features of the liver were subsequently evaluated as a damage biomarker of the hepatic tissue. Our results confirmed that 4-NP was a potent hepatotoxic agent, as 4-NP-intoxicated fish exhibited many lesions. Steatohepatitis, ballooning degeneration, sclerosing cholangitis, and coagulative necrosis of melanomacrophagecenters (MMCs) were observed. Hemosiderin, lipofuscin pigments, and proliferation of fibroblasts, kupffer cells, and telocytes were also demonstrated in the livers of 4-NP-intoxicated fish. In addition, decreased glycogen content and increased collagen deposition were observed in the hepatic tissue. Hepatocytes exhibited ultrastructural alterations in the chromatin, rough endoplasmic reticulum, smooth endoplasmic reticulum, mitochondria, lysosomes, and peroxisomes. Co-administration of 2.5% NSS to 4-NP-intoxicated fish significantly reduced these hepatotoxic effects. It nearly preserved the histological, histochemical, and ultrastructural integrity of hepatic tissue.

## Introduction

4-NP is an endocrine-disrupting element derived from a nonionic surface-active agent, 4-NP ethoxylates. The latter is widely used for domestic, agricultural, and industrial purposes^1^. Due to its lipophilic nature, 4-NP has a strong tendency to deposit in aquatic organisms^2^ until it reaches the human consumer, causing wide spread concern^1^.

4-NP is a highly hepatotoxic substance that triggers the release of hepatic enzymes into the bloodstream by causing multiple histopathological alterations^3^. It resulted in the up-regulation of apoptotic mediators, the acceleration of reactive oxidant production, and the suppression of redox stabilizer activity^4^. Numerous histopathological abnormalities in the liver following 4-NP exposure had been reported in the scholarly articles. These include hepatitis, lymphocytic cell infiltration, coagulative necrosis, nuclear changes, fatty degeneration, hepatic steatosis, disappearance of cell borders, glycogen depletion, increase in lipofuscin and hemosiderin pigments, and necrosis of endothelial cells^5–7^. Another report reveals fibrosis surrounding the vasculature and bile ductules, a dramatic increase in the size and number of MMCs, and the presence of necrotic macrophages^8^.

African catfish (*Clarias gariepinus*, Teleostei: Clariidae) is one of the most abundant species in the River Nile and its tributaries^9^. As a result of its exceptional physiological and economic characteristics from the perspectives of breeders and consumers^10^, commercial farming is a cost-effective opportunity, and investments in this field are rapidly increasing. In order to ensure safe feeding practices, significant efforts are made to enhance its health and prevent xenobiotics from reaching humans through their consumption^3,11^.

Black cumin (*Nigella sativa*, Ranun culaceae) is cultivated extensively in the tropical and subtropical zones^12^. NSS possesses a variety of redox stabilizers and cytoprotective phytochemicals, making it an excellent candidate for combating the environmental toxins^7,13^. Thymoquinone (TQ), thymol, and α-hederin are effective hepato-protective phytochemicals in *Nigella sativa* by limiting the overgeneration of reactive oxidants and inflammatory mediators, suppressing the lipid peroxidation cascade, and stimulating antioxidant network^14^. Most of the literature revealed that dietary inclusion of NSS in fish is used as a valuable strategy to reduce the hepatotoxicity of environmental pollutants by enhancing the cell membrane integrity, reductive/oxidative balance, and histo-architectural characteristics^15^. Therefore, this study aims to investigate the cytoprotective effect of NSS on the hepatic histoarchitecture, cytochemistry, and ultrastructure of 4-NP-burdened *Clarias gariepinus*.

The findings of this investigation may shed light on the importance of limiting the use and safe disposal of 4-NP as an emerging environmental toxicant, as well as the efficacy of natural products as a shield against its adverse health effects.

## Materials and methods

### Fish

Eighteen adults male *Clarias gariepinus*, weighing 351 ± 3 g, were utilized in this experiment. They appeared typical and healthy (AFS-FHS, 2003). The fish were acclimated for two weeks in aerated glass tanks containing dechlorinated tap water. Fish were fed commercial feed pellets at a rate of 5% of their body weight per day for two feedings^16^.

### Experimental design

The pre-acclimatized fish were divided into three groups (n = 6 fish per group) using a randomization technique. The first group serves as a control which did not receive any treatment except the basal diet. However, the second groups were exposed only to 4-NP (purity, 99.3%; Sigma-Aldrich, Schnelldorf, Germany) at a dose of 0.1 mg L^−1^ of aquarium water^17^. While the third group were fed a basal diet supplemented with 2.5% NSS (purchased from the Ministry of Agriculture Selling Port, Giza, Egypt) along with the same dose of 4-NP^18^. The water quality, composition of the basal diet, preparation of NSS, and the method of addition of NSS to the diet were described in our previous study^16^.

### Histological analysis

Twenty-one days after the beginning of the experiment, the fish were anesthetized using ice^19^ and livers were collected from all fish for further analysis. Pieces of liver were fixed in 10% neutral buffered formalin and Bouin’s fluid. The samples were then dehydrated in ascending ethanol concentrations, clarified in methyl benzoate, and embedded in paraffin wax. The following histological stains were applied to 5 µm-thick paraffin sections:

1. Hematoxylin and eosin for general histological examination^20^.
2. Periodic acid Schiff (PAS) stain to detect neutral mucopolysaccharides^21^.
3. Perls’ Prussian blue for ferric iron and MMCs detection^8^.
4. Crossmon’s trichrome stain for collagen fiber detection^22^.
5. Acridine orange stain for identification of necrotic hepatocytes ^23^.

### Transmission electron microscopy

Two millimeter-thick pieces of freshly sacrificed fish liver were fixed in 2.5% glutaraldehyde in phosphate buffer (pH 7.2). The fixed specimens were then washed in 0.1 M phosphate buffer and postfixed with1% osmium tetroxide. The specimens were then dehydrated in an ascending alcohol series and encased in araldite resin. Using a Reichert ultra-microtome, semi-thin sections were cut and stained with 1% toluidine blue. The ultrathin sections were then stained with uranyl acetate and lead nitrate^24,25^ and examined with a JeolJem 1200 EX Transmission Electron Microscope at the Electron Microscope Unit of Assiut University.

### Negative image analysis

Negative image analysis was carried out so that the transmission electron photomicrographs could provide greater clarity^26^.

### Ethical approval

All methods were carried out following the relevant regulations and ARRIVE guidelines. Studies were approved by the Research Ethics Committee of the Molecular Biology Research and Studies Institute (VET-22-04-R), Assiut University, Assiut, Egypt.

## Results

### The protective effects of NSS against hepatic histopathological changes in 4-NP-intoxicated *Clarias gariepinus*

**Figure 1:**
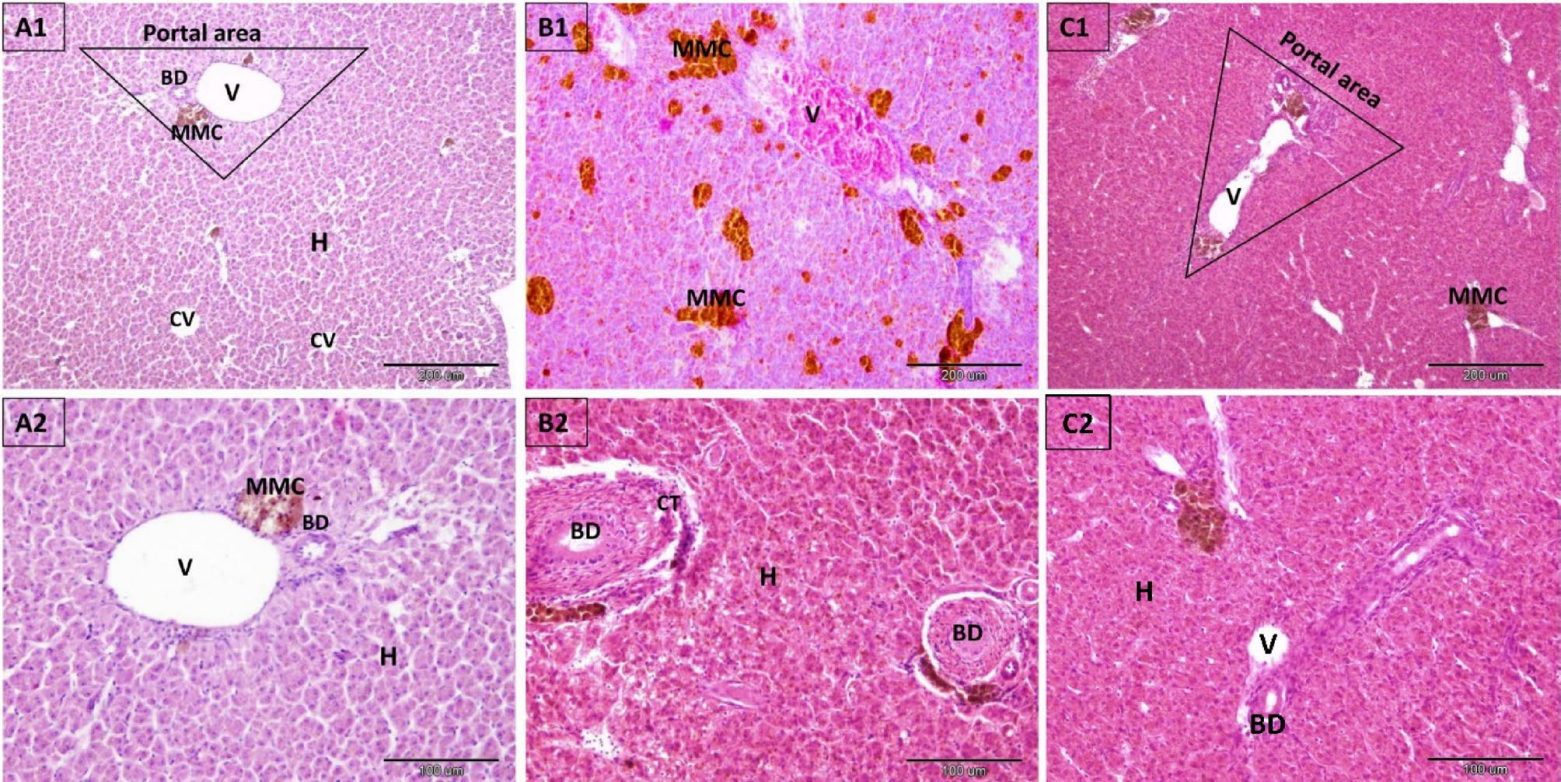
Photomicrograph of paraffin sections in the liver of control, 4-NP-intoxicated, and 2.5% NSS treated groups. (**A1**, **A2**) Control liver section depicts the normal histological architecture, which consists of a central vein (CV), hepatocytes (H), and a triangular portal area containing bile ductule (BD), portal vein (V), and melanomacrophage centers (MMC). (**B1**, **B2**) 4-NP-intoxicated liver section. (**B1**) Exhibiting a rise in melanomacrophage centers (MMC) and congested veins (V). (**B2**) Connective tissue proliferation (CT) and inflammatory cell infiltration around bile ductules (BD) (Sclerosing cholangitis). (**C1**, **C2**) Liver sections of the 2.5% NSS treated group demonstrate partially restored histological architecture and normal appearance of the portal area, portal veins (V), hepatocytes (H), and bile ductules (BD) with a reduction in the number of melanomacrophage centers (MMC) in comparison to the 4-NP-intoxicated group. Scale bar in (**A1**–**C1**) = 200 μm; (**A2**–**C2**) = 100 μm, Hematoxylin and Eosin stain.

The liver of the control fish was composed of normal hexagonal cords of hepatocytes arranged around the central vein. Hepatocytes were large in size, polygonal in shape, and contained vesicular central nuclei in homogeneous acidophilic cytoplasm. The hepatic cords were separated by blood sinusoids, which appeared to be communication channels occupied by blood cells and lined by endothelial cells and Kupffer cells (Figs. 1A_1_,A_2_, 2A,D).

**Figure 2:**
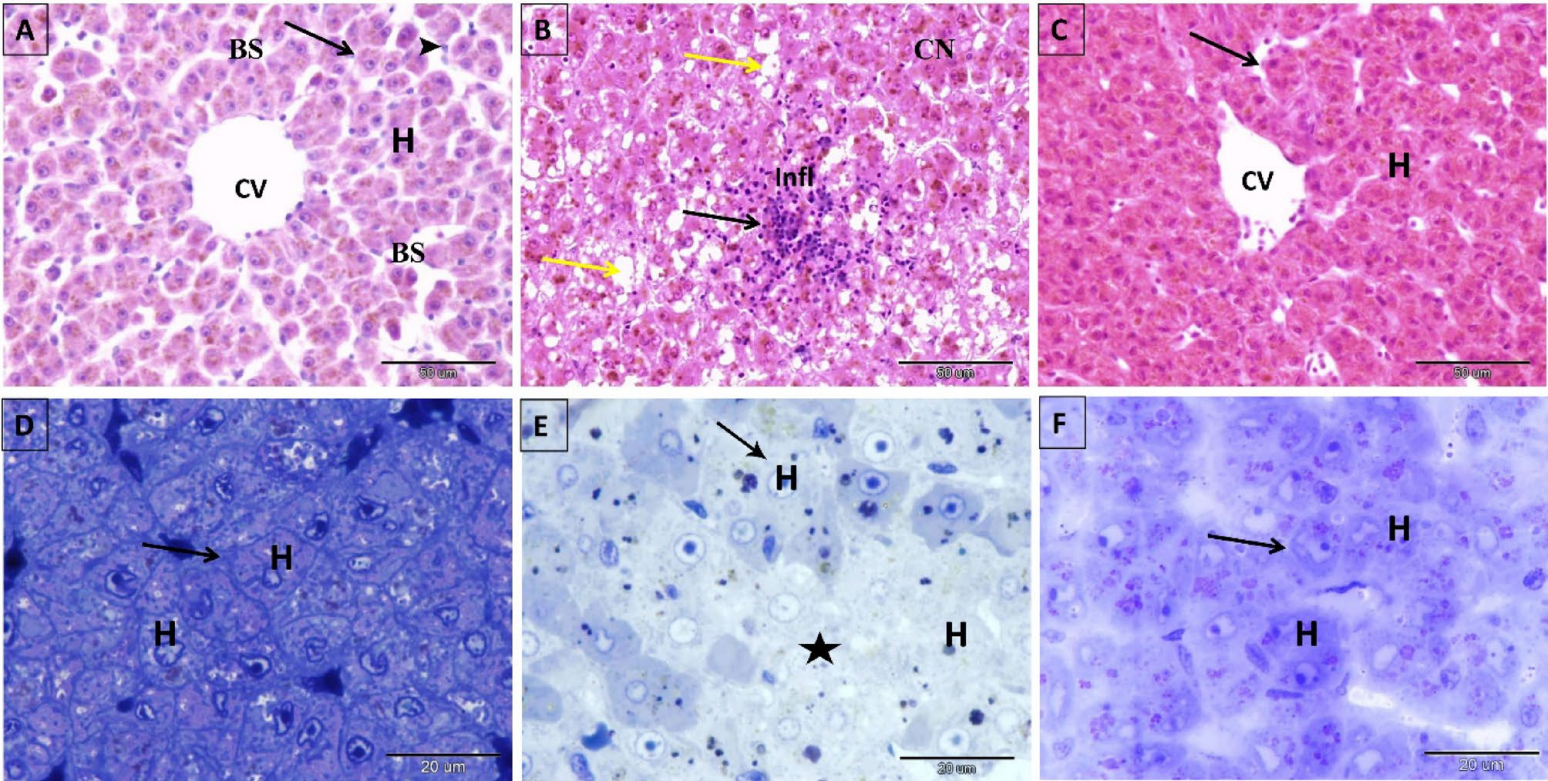
Photomicrograph of paraffin (**A**–**C**) and semi-thin (**D**–**F**) sections in the liver of control, 4-NP-intoxicated, and 2.5% NSS treated groups. (**A**) Control liver sections displaying healthy hepatocytes arranged in hepatic cords radiating from the central vein (CV) and were separated by blood sinusoids (BS), which were lined by endothelial cells (arrow) and kupffer cells (arrowhead). Hepatocytes (H) appeared as polyhedral cells with acidophilic cytoplasm and a vesicular, spherical nucleus in the center. (**B**) 4-NP-intoxicated liver section showing hepatocytes with widespread fatty degeneration as clear, small vacuoles filling the majority of hepatocytes’ cytoplasm (yellow arrow). Other hepatocytes exhibited stages of coagulative necrosis, which are characterized by deeply stained acidophilic cytoplasm and pyknotic, karyorrhexis, or nuclei loss (CN). A mononuclear inflammatory cell infiltration (infl) confirms the presence of acute hepatitis (black arrow). (**C**) The hepatic structure, hepatocytes (H), and central vein (CV) of the 2.5% NSS treated group were comparable to those of the control group, with no inflammatory cell infiltration or vacuolar fatty degeneration (arrow). (**D**) Semi-thin section of liver of control *Clarias gariepinus* displayed healthy hepatocytes (H) with a centralized nucleus and distinct cell outlines (arrow). (**E**) Semi-thin section of 4-NP-intoxicated *Clarias gariepinus* displaying cloudy swelling in the hepatocytes (H) with clear pale cytoplasm (arrow) and area of ballooning degeneration (star) containing hepatocytes with faintly stained cytoplasm, absence of cell outlines, and eccentric, pyknotic, or absent nuclei. (**F**) Semi-thin section of the liver of 2.5% NSS treated *Clarias gariepinus* demonstrates an improvement in the histological appearance of the liver, with more healthy, deeply stained hepatocytes (H) containing round vesicular nuclei and distinct cell outlines (arrow). Scale bar in (**A**–**C**) = 50 μm and stained with Hematoxylin and Eosin stain; (**D**–**F**) = 20 μm and stained with Toluidine blue stain.

Following exposure to 0.1 mg L^−1^ 4-NP for 21 days, liver sections exhibited a loss of hexagonal architecture. Most hepatocytes displayed coagulative necrotic changes as the disintegration of most cytoplasmic contents with faintly stained cytoplasm and pyknotic nuclei or loss of nuclei (Figs. 1B_2_, 2B). Other hepatocytes exhibited vacuolar fatty degeneration with eccentric nuclei (macro and microvesicular steatosis) in their cytoplasm (Fig. 2B). Blood stagnation was also observed in the dilated sinusoids, central veins, and portal veins (Fig. 1B_1_). The infiltration of mononuclear inflammatory cells was also observed in the necrotic areas, indicating the presence of hepatitis (Fig. 2B). Coadministration of 2.5% NSS improved and partially restored the histological structures of the liver of 4-NP-intoxicated fish, Most of the hepatocytes had pink-stained cytoplasm and lacked clear fat vacuoles (Figs. 1C_1_,C_2_ & 2C).

Examining semi-thin sections stained with toluidine blue revealed that the liver of the 4-NP-exposed group contained large areas of ballooning degeneration (cloudy swelling) that consisted of hepatocytes with faintly stained cytoplasm, absence of cell outlines, and nuclei that are eccentric, pyknotic, or absent (Fig. 2E) compared to the liver of  the unexposed group (Fig. 2D). Coadministration of 2.5% NSS improved the histological appearance of hepatocytes manifested by the presence of stained cytoplasm and nuclei in the center, resembling the control group (Fig. 2F).

### The protective effects of NSS on the amount of the hepatic MMCs in 4-NP-intoxicated *Clarias gariepinus*

**Figure 3:**
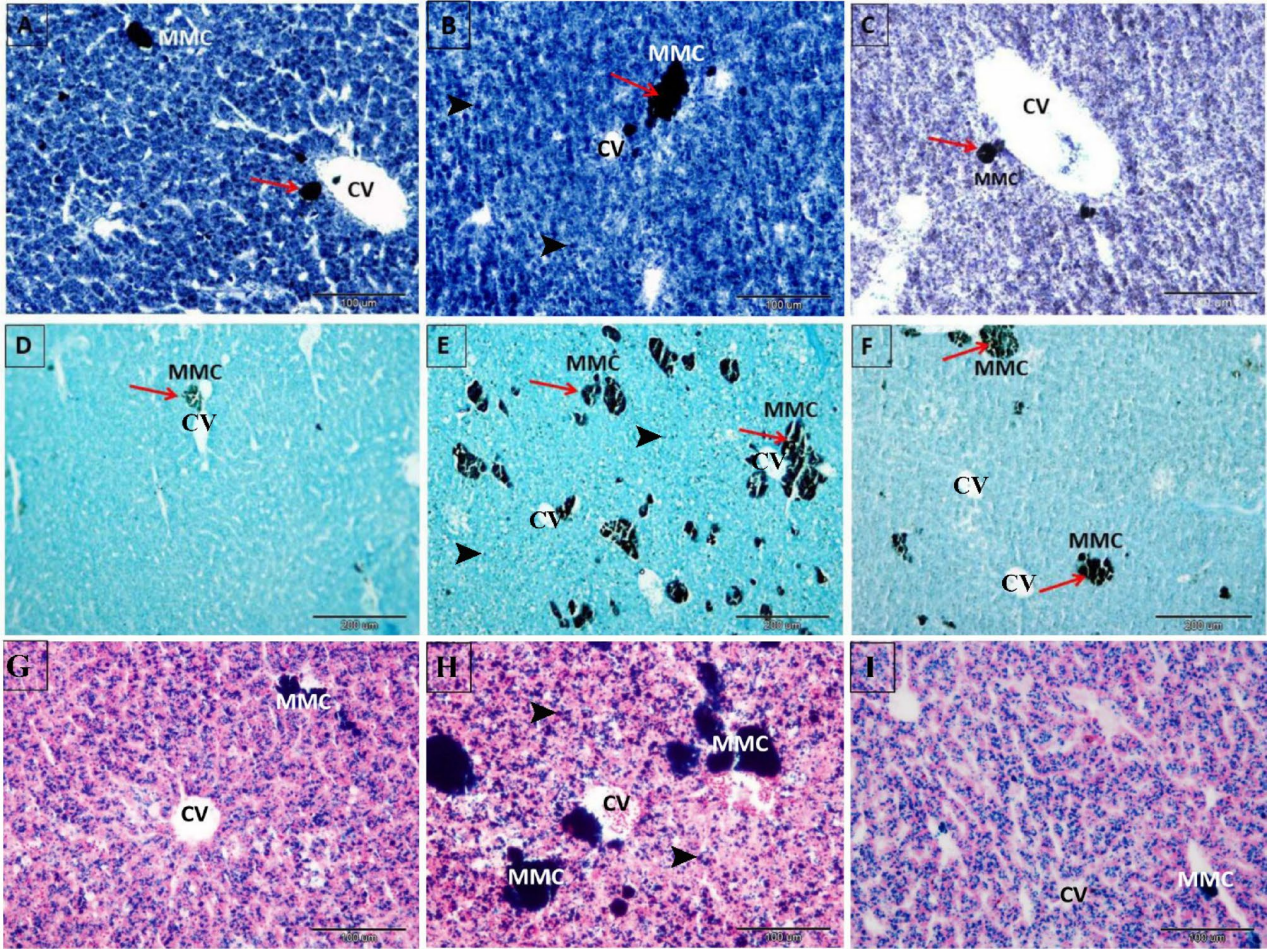
Photomicrograph of paraffin-embedded sections illustrating the hepatoprotective effects of NSS against 4-NP-induced hepatic MMCs disturbance in *Clarias gariepinus*. (**A**) Liver of control *Clarias gariepinus* showing a less amount of Sudan black B-positive MMCs (arrow). (**B**) Liver of 4-NP-intoxicated *Clarias gariepinus* showing an increase in the amount of Sudan black B-positive MMCs (arrow). (**C**) Liver of 2.5% NSS treated *Clarias gariepinus* showing a fewer Sudan black B-positive MMCs. Note the central vein (CV) and the abundant Sudan black-positive lipofuscin pigments (arrowheads) that filled the hepatocytes of 4-NP-intoxicated *Clarias gariepinus* compared to the control group and the NSS + 4-NP treated group. (**D**) The liver of control *Clarias gariepinus* contains a fewer Nile blue-positive MMCs (arrow). (**E**) The liver of 4-NP-intoxicated *Clarias gariepinus* demonstrates an increase in Nile blue-positive MMCs (arrow) surrounding the central vein (CV). (**F**) Liver of 2.5% NSS treated *Clarias gariepinus* showing a decline in the amount of Nile blue-positive MMCs (arrow) similar to control liver. Note the central vein (CV) and the abundant Nile blue-positive lipofuscin pigments (arrowheads) that filled the hepatocytes of 4-NP-intoxicated *Clarias gariepinus* compared to the control and NSS + 4-NP treated groups. (**G**) The liver of the control group displayed a small number of Prussian blue-positive MMCs of small size. (**H**) The 4-NP-intoxicated group’s liver contains numerous large Prussian blue-positive MMCs. (**I**) The liver of the 2.5% NSS + 4-NP-treated group reveals a small number of Prussian blue-positive MMCs of small size. Note the central vein (CV) and the abundant Prussian blue-positive hemosiderin pigments (arrowheads) that filled the hepatocytes of 4-NP-intoxicated *Clarias gariepinus* compared to the control group and the NSS + 4-NP treated group. Scale bar in (**A**–**C**, **G**–**I**) = 100 μm; (**D**–**F**) = 200 μm. (**A**–**C**) were stained with Sudan black B and Haematoxylin stain, (**D**–**F**) were stained with Nile blue stain, and G, H and I were stained with Perls’ Prussian blue stain.

The amount (size and number) of Sudan black, Nile blue, Prussian blue, and PAS-positive hepatic MMCs increased in the 4-NP-intoxicated group (Fig. 3B,E,H, respectively). These hepatic MMCs were primarily gathered around the clogged central veins, bile ductules, and portal veins in the portal regions. The hepatocytes of *Clarias gariepinus* intoxicated with 4-NP also displayed abundant Sudan black and Nile blue-positive lipofuscin pigments (Fig. 3B,E, respectively) as well as large numerous Prussian blue-positive hemosiderin pigments (Fig. 3H).

Coadministration of 2.5% NSS improved these characteristics (Fig. 3C,F,I), which resembled those of the control group (Fig. 3A,D,G).

### The protective effects of NSS on the hepatic collagenous fiber proliferation and glycogen content in 4-NP-intoxicated *Clarias gariepinus*

Fibrous tissue proliferation around the congested veins and bile ductules in the portal area and inflammatory cell infiltration around the fibrotic bile ductules indicated the presence of sclerosing cholangitis in the liver of 4-NP-intoxicated *Clarias gariepinus* (Figs. 1B2 and 4B). Conversely, fibrous connective tissue proliferation around the central veins and bile ductules in the NSS + 4-NP treated group (Figs. 1C2, 4C) was less than in the 4-NP-intoxicated group and resembled the normal non-exposed group (1A_1_, 1A_2_& 4A).

**Figure 4:**
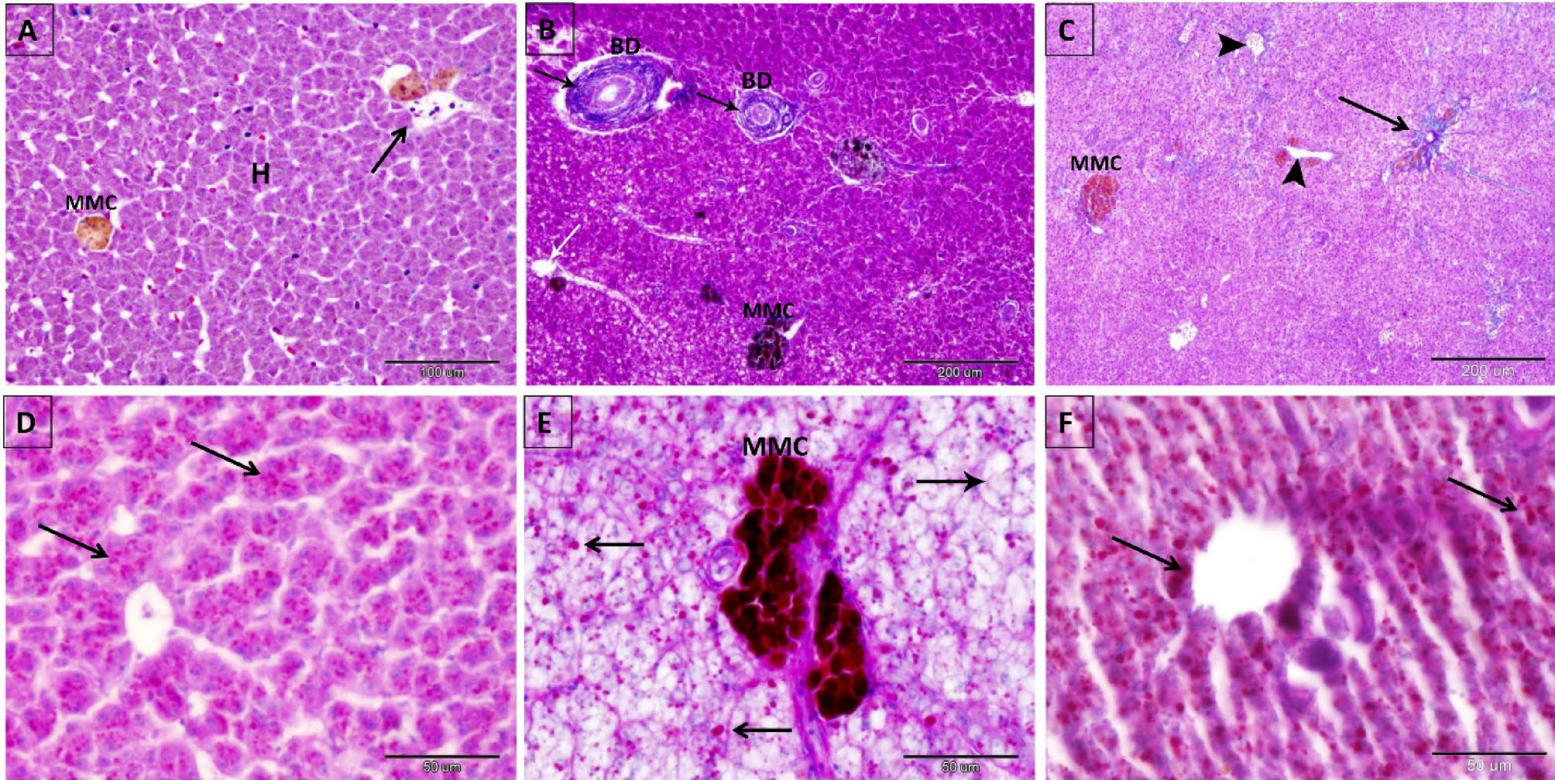
Photomicrograph of paraffin sections in the liver of control, 4-NP-intoxicated, and 2.5% NSS treated groups. (**A**, **D**) Liver of control *Clarias gariepinus*. (**A**) Displaying normal hepatic structure and hepatocytes (H) and minimal connective tissue around the central veins (Arrow), (**D**) Showing the presence of numerous fine PAS-positive glycogen granules in the cytoplasm of hepatocytes. (**B**, **E**) Liver of 4-NP-intoxicated *Clarias gariepinus*. (**B**) Showing proliferation of connective tissue around the bile ductule (BD) (sclerosing cholangitis) in the portal area (black arrow) and around the central veins (white arrow). (**E**) Demonstrating fatty change (macrovesicular steatosis) of hepatocytes with depletion in the PAS-positive glycogen granules (arrow). Note the PAS-positive MMCs. (**C**, **F**) Liver of 2.5% NSS treated *Clarias gariepinus*. **C:** Showing partial decrease in the amount of connective tissue in the portal area (arrow) and around the central veins (arrowheads) compared to 4-NP-treated *Clarias gariepinus*. (**F**) Showing hepatocytes partially restored PAS-positive glycogen granules in the cytoplasm (arrow). Scale bar in (**A**) = 100 μm; (**B**, **C**) = 200 μm; (**D**, **E**), F = 50 μm. (**A**–**C**) were stained with Crossmon’strichrome stain. (**D**–**F**) stained with periodic acid Schiff reagent (PAS) and Hematoxylin stain.

Numerous glycogen granules in the cytoplasm of hepatocytes were diminished in the 4-NP-exposed group compared to the non-exposed control group (Fig. 4D,E). Coadministration of 2.5% NSS partially restored hepatocyte glycogen content (Fig. 4F).

### The protective effect of NSS against 4-NP-induced hepatic DNA damage

**Figure 5:**
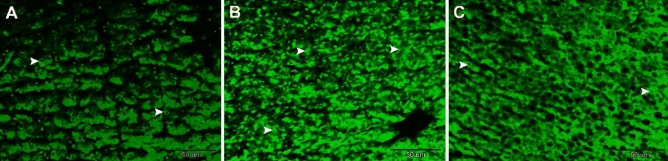
Fluorescence photomicrograph of paraffin-embedded sections illustrating the hepatoprotective effects of NSS against 4-NP-induced hepatic damage in *Clarias gariepinus*. (**A**) Hepatocytes of the control group’s liver have healthy nuclei (arrowheads). (**B**) Numerous hepatocytes are visible in the liver of the 4-NP-intoxicated group containing necrotic and broken nuclei (arrowheads) **C:** The NSS + 4-NP-treated group’s liver contains hepatocytes with healthy nuclei (arrowheads). The necrotic nuclei (DNA) stained with acridine orange appeared as orange spots under a fluorescence microscope. Scale bar = 50 μm, acridine orange stain.

The liver of the 4-NP-intoxicated group exhibited necrotic hepatocytes and damaged nuclei (Fig. 5B). While the liver of NSS + 4-NP treated group exhibited hepatocytes with healthy nuclei (Fig. 5C), nearly identical to the control liver (Fig. 5A). When stained with acridine orange and viewed through a fluorescence microscope, the damaged DNA appeared as orange spots.

### The protective effects of NSS against the hepatic ultrastructural changes in 4-NP-intoxicated *Clarias gariepinus*

**Figure 6:**
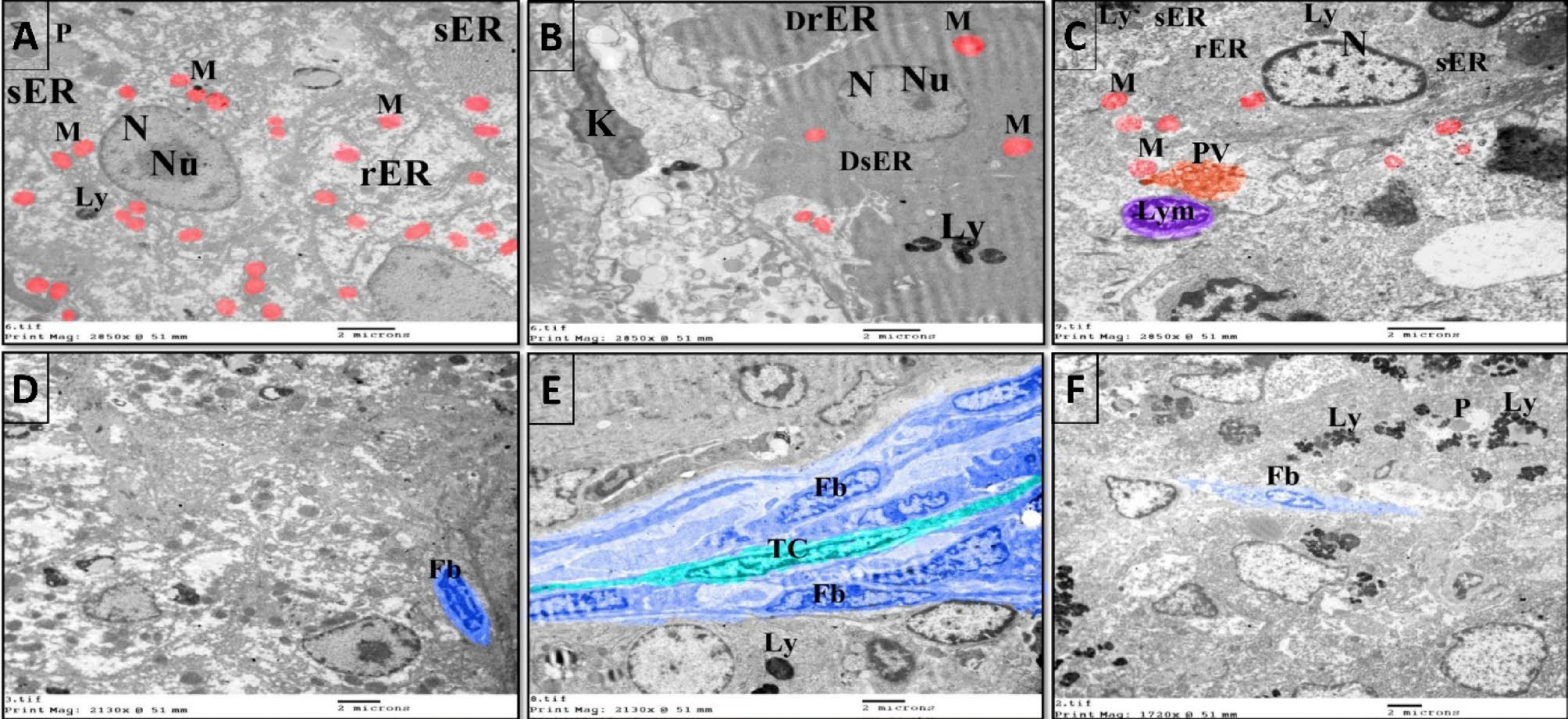
Colored transmission electron photomicrographs illustrating the hepatoprotective effects of NSS against 4-NP-induced hepatotoxicity in *Clarias gariepinus* using colored transmission electron micrographs. (**A**) Hepatocyte of the control group displaying euchromatic nucleus (N) with distinct nucleolus (Nu), well-developed rough endoplasmic reticulum (rER), well-developed smooth endoplasmic reticulum (sER), abundant mitochondria (M), and few lysosomes (Ly) and peroxisomes (P). (**B**) Hepatocyte of the 4-NP-intoxicated group showing nucleus (N) with decreasing amount of heterochromatin and degenerated nucleolus (Nu), degenerated rough endoplasmic reticulum, degenerated smooth endoplasmic reticulum, Kupffer cells (K), few mitochondria (M), and lysosomes (Ly). (**C**) Hepatocyte of the NSS + 4-NP-treated group displaying euchromatic nucleus (N), nearly healthy rough endoplasmic reticulum (rER), smooth endoplasmic reticulum (sER), mitochondria (M), phagocytic vacuole, and lysosomes (Ly). Note the lymphocyte (Lym). (**D**) Liver of the control group showing few fibroblasts (Fb). (**E**) Liver of 4-NP-intoxicated group showing numerous fibroblasts (Fb). Note the telocyte (TC). (**F**) Liver of NSS + 4-NP treated group showing few fibroblasts (Fb). Note the numerous lysosomes (Ly) and peroxisomes (P) on the hepatocytes.

**Figure 7:**
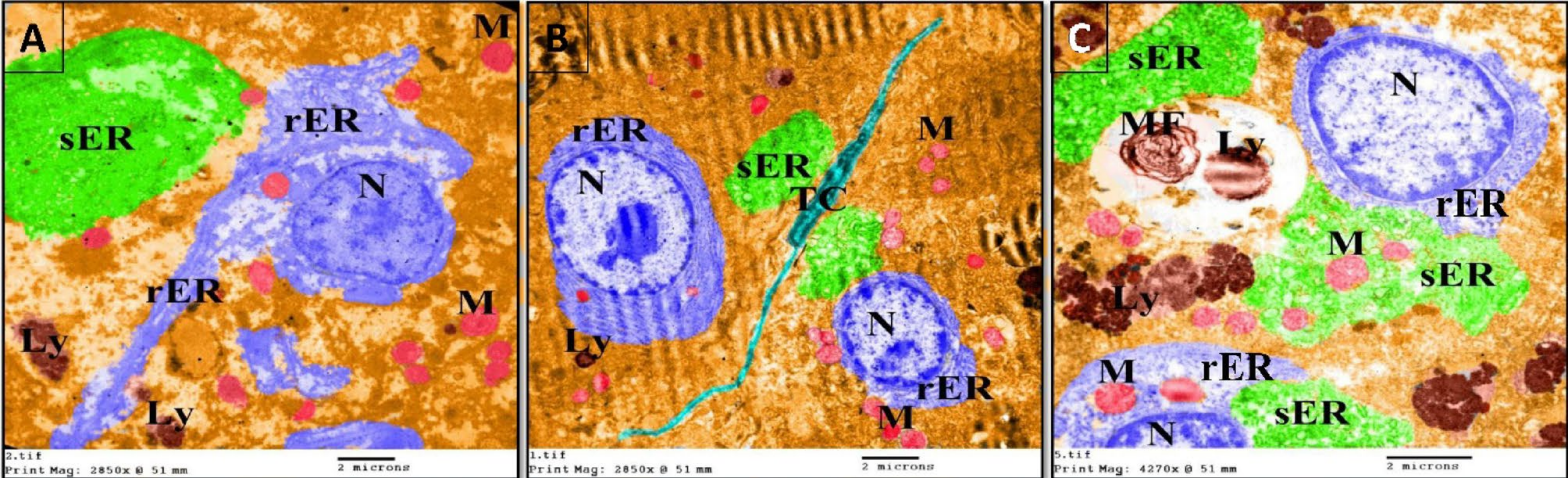
Colored transmission electron photomicrographs illustrating the hepatoprotective effects of NSS against 4-NP-induced hepatotoxicity in *Clarias gariepinus*. (**A**) Hepatocyte of the control group with euchromatic nucleus (N), well-developed rough endoplasmic reticulum (rER), well-developed smooth endoplasmic reticulum (sER), abundant mitochondria (M), and few lysosomes (Ly). (**B**) Hepatocyte of the 4-NP-intoxicated group displaying euchromatic nucleus (N), a small amount of rough endoplasmic reticulum (rER), smooth endoplasmic reticulum (sER), small sized mitochondria (M), and a small number of lysosomes (Ly). Note the telocyte (TC). (**C**) Hepatocyte of the NSS + 4-NP-treated group displaying euchromatic nucleus (N), rough endoplasmic reticulum (rER), smooth endoplasmic reticulum (sER), mitochondria (M), and myelin figure (MF), as well as numerous lysosomes (Ly).

The hepatocytes of the control group had euchromatic nuclei with distinct nucleoli, well-developed rough and smooth endoplasmic reticulum, abundant mitochondria, few lysosomes, and peroxisomes.  Few fibroblasts were observed in the hepatic tissue of the control group (Figs. 6A,D, 7A). On the other hand, many of the hepatocytes in the 4-NP-intoxicated group exhibited decreasing heterochromatin and degenerated nucleoli, rough endoplasmic reticulum, and smooth endoplasmic reticulum. A few mitochondria and lysosomes were also demonstrated in the hepatocytes of the 4-NP-intoxicated group (Figs. 6B,E, 7B). In addition, kupffer cells, fibroblast proliferation and telocytes were observed in the liver tissue of 4-NP-intoxicated fish (Figs. 6B,E, 7B). In contrast, the hepatocytes of the NSS + 4-NP-treated group exhibited euchromatic nuclei, nearly healthy rough and smooth endoplasmic reticulum and mitochondria. In addition, the liver of the NSS + 4-NP-treated group contained lymphocytes, peroxisomes, phagocytic vacuole, and a significant number of lysosomes (Figs. 6C,F, 7C).

**Figure 8:**
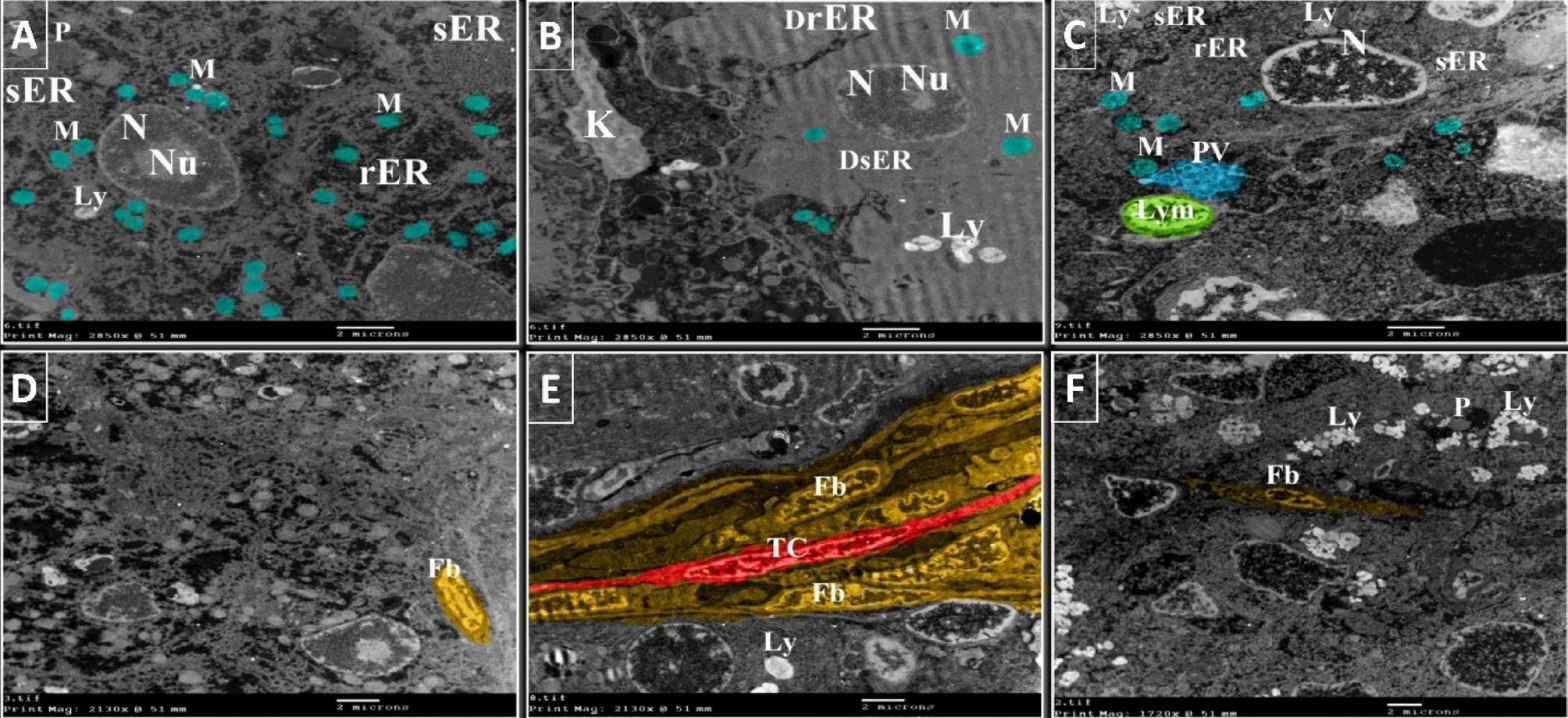
Negative images of the photomicrographs shown in Fig. 6.

The colored transmission electron photomicrographs (Figs. 6, 7) and negative images (Fig. 8) of Fig. 6 were employed to determine the cytoprotective effect of NSS against 4-NP-induced hepatocellular damage in *Clarias gariepinus.*

## Discussion

*Nigella sativa* is fully armed with redox stabilizers and cytoprotective constituents^11^ giving a driving force for the scientific community to utilize it as an effective approach against several aquatic contaminants. One of the most dangerous compounds agents is 4-NP owing to resistance to biodegradation, wide prevalence in the ecosystem, and high probability to reach to the consumers^27,28^. It represents a main risk hazard as it induces a battery of toxicological aspects, including reproductive dysfunction^29^, immunosuppressive, and hepato- and nephrotoxic impacts^3^. Thus, this study is designed to highlight the potential protective effects of *Nigella sativa* against the histo-architectural and ultrastructural changes in the liver of 4-NP-intoxicated *Clarias gariepinus*.

The histological changes in the livers of 4-NP-exposed *Clarias gariepinus* are consistent with those described in previous scholarly works^7,30^. By increasing pro-inflammatory cytokines^31^ and chemotactic proteins, 4-NP could be associated with inflammatory cell infiltration in the hepatic tissues^32^. The activation of pro-apoptotic markers may be one of the negative effects of inflammation^33^. Abd-Elkareem et al.^7^ reported that the hepatocytes of *Clarias gariepinus* contain vacuolated cytoplasm under 4-NP stress. This outcome can be explained by the overexpression of the lipogenic enzyme genes and the downregulation of the transcription factors involved in fatty acid oxidation^33^. These metabolic changes result in the deposition of triglycerides, which are removed during tissue processing by organic solvents, leaving hollow spaces unstained^7^. Aggregation of misfolded proteins and expansion of the endoplasmic reticulum lumen occurred when the hepatocytes exposed to peroxidative insult^34^.

The morphology and content of pigments in MMCs are sensitive to various internal and external factors^35^; therefore, we used staining to monitor their differential responses to 4-NP contamination. As previously observed, the content of Nile blue-positive MMCs increased significantly^36,37^, reflecting its ability to neutralize toxicants^38^ and quench reactive oxidants^39^. The increase in melanin is a morphological response triggered by reactive species overloading^36^, to counteract them, as well as the harmful derivatives that emerged from the breakdown of cellular components^40^. In multiple ecotoxicological studies, hepatic melanin is thus a sensitive biomarker for aquatic pollution^8,41^. Numerous Sudan black and Nile blue-positive lipofuscin pigments were present in the hepatocytes of 4-NP-intoxicated *Clarias gariepinus*, similar to recent findings^37^. Lipofuscin is the final consequence of the accumulation of highly oxidized cross-linked proteins, indicating that the cellular proteolytic capacity falls below the sub-threshold level required to manage the redox disturbance^42^. Overproduction of free radicals and induction of cell death^7,36^ may account for the increased lipofuscin amount in MMCs following the exposure to 4-NP. Lipofuscin triggers a vicious redox instability and apoptosis cycle by increasing caspase-3 activity^43^ and producing free radicals^44^. Numerous hepatocytes with necrotic and damaged nuclei were discovered in the 4-NP group, confirming this fact. As an adaptive response to 4-NP-induced tissue destruction, the abundance of lipofuscin reflects the upregulation of phagocytic activity in MMCs^7^.

Depletion of intrahepatic glycogen content in response to 4-NP exposure is comparable to that observed in *Clarias gariepinus*^7^ but not in Italian newt (*Lissotrito nitalicus*)^36^. This contradiction results from differences in fish species, doses of 4-NP, and duration of the intervention. Long-term exposure to 4-NP disrupts the insulin signaling downstream pathway triggering insulin resistance and alters the carbohydrate metabolizing machinery due to oxidative damage to the liver^45^.

The upregulation of collagen expression is responsible for the excessive fibrosis surrounding the central veins and bile pathway in the 4-NP-exposed group^46^. The impeded intrahepatic perfusion, secondary to fibrosis, stimulates advanced fibrogenesis and subsequent portal hypertension^47^.

The genotoxicity of 4-NP is caused by the promotion of redox disequilibrium^48^, increase in the transcript level of pro-apoptotic regulators^33^, inhibition of the endoplasmic reticulum Ca^2+^ pump^49^, and loss of mitochondrial membrane electricity^50^.

Bernabò et al.^36^ found that the ultrastructural changes in hepatocytes following the 4-NP intoxication are similar to those found in *Lissotrito nitalicus*. It is common knowledge that the nucleolus is the RNA processing center and ribosome factory^51^. Thus, the degeneration in the nucleoli of hepatocytes caused by 4-NP supplementation reveals a slowdown in translating genetic codes into polypeptide sequences. This slowdown in translating genetic codes resulting in a decrease in the cell’s ability to produce structural and functional proteins, ultimately leading to alterations in the hepatic microenvironment.

Kupffer cells appeared in the 4-NP group, indicating a trial of the hepatic protective device to stimulate the phagocytic activity of sinusoidal cells in order to detoxify 4-NP and its degradation intermediates^52^. The hypertrophy and hyperplasia in the MMC of 4-NP group are consistent with previous observations in 4-NP-intoxicated *Clarias gariepinus* and goldfish (*Carassius auratus*)^8,37^. This response is considered a compensatory adaptation^8^ to a suppressed innate immunity, as it is closely linked to pollutant loading^53^.

Bernabò et al.^36^ described the degeneration in the rough endoplasmic reticulum in response to exposure to various chemical toxins, including 4-NP. The remarkable appearance of an increased number of cytosolic lipid bodies is associated with damage to the rough endoplasmic reticulum, indicating a potential causative link^54^. This link may result from a decrease in protein synthesis, which inhibits the consumption of lipids in lipoprotein aggregation^36^.

The lack of mitochondria indicates that 4-NP can directly inhibit ATP synthesis, resulting in bioenergetic deficiency^55^. Due to their lipophilic nature, the endocrine disrupting chemicals interact with the hydrophobic lipid matrix of membranes disrupting the phospholipid vesicles^56^. The decrease in mitochondrial biogenesis may be attributable to a change in peroxisome proliferator-activated receptor-γ coactivator-1α^33^; a co-transcriptional regulation factor responsible for this process by interacting with numerous transcription proteins. As mitochondria are especially susceptible to oxidative stress, an excess of free radicals could be a leading cause of mitochondrial damage^57^. DNA mutations, respiratory chain damage, membrane permeability disruption, and mitochondrial defense suppression are induced by mitochondrial stress^58^.

The presence of telocytes in 4-NP-intoxicated liver tissue indicates attempts to promote tissue regeneration and repair, slow down abnormal stimulation of immune cells and fibroblasts, and reduce the matrix architecture transformation during fibrosis^59^.

According to a previous report, the normalization of hepatic histo-architecture following the administration of NSS to 4-NP-intoxicated fish is attributable to an improvement in the hepatic antioxidant defensive network^15^. NSS has an abundance of redox stabilizers, including thymoquinone, flavonoids, and terpenoids^11^. NSS boosts the transcript level of redox stabilizers^60^, and shifts the cell fate decisions to pro-survival events^61^. By elevating proliferating cell nuclear antigen, TQ stimulates cell multiplication, thereby enhancing the ability of cell to regenerate after tissue injury^62,63^.

In our study, NSS supplementation protected the liver from excessive fibrosis and restricted the inflammatory infiltration similar to that observed in the glomeruli of 4-NP-intoxicated *Clarias gariepinus*^13^ and the myocardium of lipopolysaccharide-intoxicated rats^64^. This result can be explained by the ability of 4-NS to reduce the fibrogenic and proinflammatory mediators^65,66^.On the genetic level, TQ inhibits the expression of profibrotic and nuclear factor Kappa-B^67,68^.

The restoration of hepatic glycogen content in the NSS + 4-NP-treated group is in the same line as that observed in Rohu (*Labeorohita*) fingerlings exposed to diethyl phthalate^15^, secondary to stimulation of insulin release which promotes glycogenesis^69^.

NSS intervention decreased the number of hepatic MMCs and the characteristics of autophagy in 4-NP-intoxicated fish, paralleling the reduction observed in the glomeruli of nephrotoxic *Clarias gariepinus*^13^. The immune response of MMCs was normalized due to the motivation in the detoxification and biotransformation pathways of xenobiotics^70^ and the limitation in generating reactive oxidants^11^.

The appearance of euchromatic nuclei indicates the restoration of active transcription, paving the way for the resumption of normal cellular synthetic apparatus^71^. This action is necessary to restore hepatocyte viability and repair damaged hepatocytes^72^. The presence of peroxisome in the 4-NP + NSS group contributes to the maintenance of redox homeostasis^73^, elimination of oxidizing proteins^74^, and decrease in the likelihood of lipid peroxidation^75^. The presence of phagocytic vacuoles in the hepatic tissue of the 4-NP + NSS group indicates the activation of immune defensive mechanisms to counteract the cytotoxicity of 4-NP metabolites through electrophile and oxidant detoxification^76^. The lysosome is a necessary prerequisite for autophagy. This process promotes cell survival by removing damaged organelles and protein aggregates and promoting bioenergetic balance^77^. Thus, the abundance of lysosomes may be associated with the effort to repair and regenerate the attacked cell and remove cell debris caused by 4-NP-induced oxidative damage. This response was first observed in the epithelial cells of proximal tubule^23^.

The role of NSS as a useful alternative in preventing mitochondrial degeneration^62^ and endoplasmic reticulum stress^78^ signifies the restoration of the healthy characteristics of these organelles. This result may be due to down-regulation of apoptotic cascade and quenching of lipid peroxidation products, maintaining the normal membrane permeability of the cell organelles^78^.

The hepatocytes of 4-NP + NSS group displayed healthy nuclei. The genoprotective potential of NSS may be mediated by TQ, which increases the transcript levels of Bcl2, decreases the transcript levels of caspase-3 and Bax^79^, and inhibits oxidative stress-induced DNA fragmentation^80^.

In conclusion, NSS is an effective hepato-protective agent against the cytotoxicity of 4-NP in *Clarias gariepinus* by preserving the histological, histochemical, and ultrastructural integrity of the hepatic tissue. In order to evaluate the effects of these cytological improvements on the liver functions, additional research are required.

## Data Availability

All data generated or analyzed during this study are included in the research article.
